# Polymer Binders: Characterization and Development toward Aqueous Electrode Fabrication for Sustainability

**DOI:** 10.3390/polym13040631

**Published:** 2021-02-20

**Authors:** Aleksander Cholewinski, Pengxiang Si, Marianna Uceda, Michael Pope, Boxin Zhao

**Affiliations:** Department of Chemical Engineering, Waterloo Institute for Nanotechnology, Institute for Polymer Research, Centre for Bioengineering and Biotechnology, University of Waterloo, Waterloo, ON N2L 3G1, Canada; aacholew@uwaterloo.ca (A.C.); p2si@uwaterloo.ca (P.S.); muceda@uwaterloo.ca (M.U.); michael.pope@uwaterloo.ca (M.P.)

**Keywords:** binder, energy storage, lithium ion battery, aqueous electrode, carboxymethylcellulose, battery characterization

## Abstract

Binders play an important role in electrode processing for energy storage systems. While conventional binders often require hazardous and costly organic solvents, there has been increasing development toward greener and less expensive binders, with a focus on those that can be processed in aqueous conditions. Due to their functional groups, many of these aqueous binders offer further beneficial properties, such as higher adhesion to withstand the large volume changes of several high-capacity electrode materials. In this review, we first discuss the roles of binders in the construction of electrodes, particularly for energy storage systems, summarize typical binder characterization techniques, and then highlight the recent advances on aqueous binder systems, aiming to provide a stepping stone for the development of polymer binders with better sustainability and improved functionalities.

## 1. Introduction: Binders and Electrodes for Energy Storage Systems

**Figure 1:**
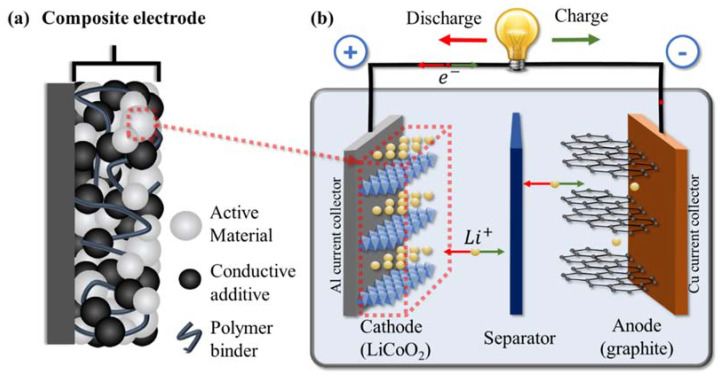
Schematic for (**a**) a composite electrode including the active material, conductive additive, and polymeric binder; (**b**) a full lithium-ion battery (LIB) with LiCoO_2_ used as the active material for the cathode and graphite anode during discharge (with reactions shown occurring within a crystallite of active material).

Electrochemical energy storage systems, such as batteries, play an important role together with renewable energy sources for creating a greener and more sustainable future. When developing new solutions and improving battery performance, the primary focus has been toward electrode active materials and electrolytes. Binders, on the other hand, have received comparatively little attention, although recent reviews have begun to highlight their importance, especially in high-capacity battery systems [1,2,3,4]. While they only make up a small portion of the electrode material (typically 2–5% of the mass in commercial electrodes), binders play multiple important roles in battery performance. First, they help to disperse the other components in solvent during the fabrication process (with some also acting as a thickener), enabling a homogeneous distribution [5,6]. Second, they hold together the various components of energy storage devices, including the active components, any conductive additive, and the current collector, ensuring all these pieces are kept in contact [1,7] (Figure 1a shows a schematic for a composite electrode with binder interacting with the various components). Through chemical or physical interactions, the binder bridges these separate components, keeping them together and ensuring mechanical integrity of the electrode without significantly impacting electronic or ionic conductivity. Third, they often act as an interface between the electrode and electrolyte. In this role, they can protect the electrode from corrosion or the electrolyte from depletion while facilitating ion transport across this interface [8,9].

With all the roles that binders play in an electrode (and the overall battery), there are many different properties that are desirable in a good binder. To better understand these desired properties, it is useful to examine the structure of a typical battery. Lithium-ion batteries (LIB) consist of an anode and cathode separated via a porous membrane separator and/or electrolyte (which may be liquid or solid). The electrodes and electrolyte are enclosed within a protective casing and are externally connected through circuitry. A simplified cell schematic can be seen in Figure 1b where lithium cobalt oxide (LiCoO_2_) and graphite are the active materials used in the cathode and anode, respectively. In this system, the cathode is composed of layered materials with freely moving lithium ions that travel between the anode and cathode during charge/discharge. The ions will move within the layered material where the storage of lithium ions is enabled via reduction/oxidation of the transition metal (in this case, Co^4+^/Co^3+^). The electrodes themselves are comprised of active, conductive, and binding materials cast onto a metallic current collector. The conductive material compensates for the poor electronic conductivity, thereby facilitating charge transfer and improving electrode kinetics. Binders interact with many of these components, each of which add requirements to maintain the stability and performance of the cell.

**Figure 2:**
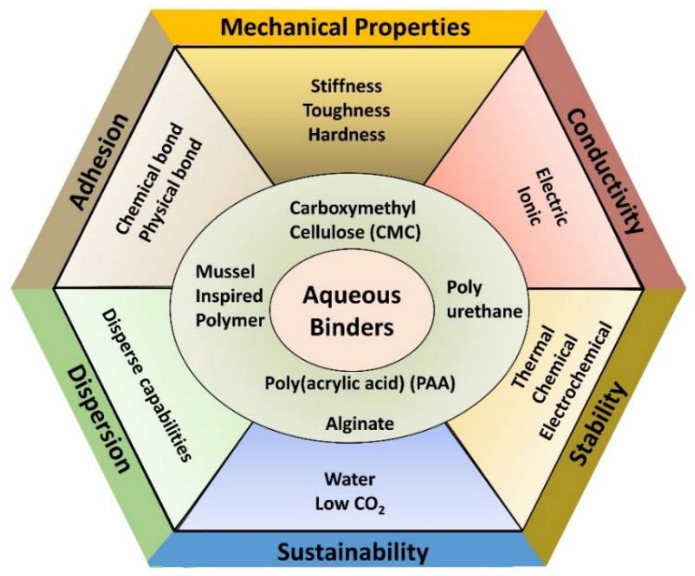
Requirements and desired properties of binders in energy storage devices. Aqueous binders can address many of these requirements, with several examples shown in the center.

The battery structure highlights the importance of several properties for polymer binders, which are summarized in Figure 2. Mechanical properties, which include the stiffness, toughness, and hardness of the binder as well as its adhesion to the other components, are important for the electrode to withstand the forces that result from the expansion and contraction of active materials during charge/discharge cycles. Thermal properties, particularly thermal stability, are also important, both for the high temperatures commonly used for curing and drying in electrode fabrication as well as for the operation of the final device in various conditions. Similarly, chemical and electrochemical stability are essential binder properties to allow them to function for long periods and over numerous cycles without degradation of the energy storage system. The binder should not react with any other components or intermediates formed during operation and should remain stable at the high and low potentials experienced by the cathode and anode, respectively. Good dispersive capabilities are also extremely helpful for binders to possess and can help evenly distribute the other components during fabrication. These depend on properties of the polymer chains, including the presence of charges, their density, and chain flexibility, which all play a role in the electrostatic repulsion and resisting depletion flocculation [6,10]. While not necessary, binders would ideally support electrical and ionic conductivity in the energy storage device. Electrical conductivity can primarily be achieved in polymers through a conjugated network and free charge carriers and could replace the need for a conductive additive to electrodes. Along with electrical conductivity, ionic conductivity is important for electrode performance, and it is affected by the structural and mechanical properties of the polymer, such as crystallinity and viscosity. Ionic conductivity can also be achieved through specific mechanisms from the presence of functional groups, such as Li ion hopping [11,12].

## 2. Binder Characteristics and Performance Evaluation

A large number of characterization techniques are commonly used to evaluate binders and their performance. These include chemical analysis, the characterization of mechanical properties, and various electrochemical tests. The following section will review many of these techniques and how they can be utilized to investigate binders and their performance, with Table 1 providing a summary of these techniques and their applications.

### 2.1. Chemical and Thermal Characterization for Stability

Fourier transform infrared spectroscopy (FTIR) can be used to confirm the chemical structure of binders. When chemical compounds are irradiated with wavelengths of infrared light, functional groups can generate vibrations via a variety of ways such as stretching or bending. These vibrations and their intensity result in an FTIR spectrum through Fourier transform. Since most binders’ mechanical properties are derived from chemical crosslinking, new peaks appear in the FTIR spectrum indicating the formation of covalent bonds between the raw binder materials. For example, in a hybrid binder of PU/PAA (polyurethane/poly(acrylic acid)), PU shows stretching peaks at 1663 and 3330 cm^−1^ corresponding to the –O–C=O– and –OH groups, and PAA shows a stretching peak at 1707 cm^−1^ corresponding to the –COOH group. After thermal crosslinking of PU and PAA, a new peak appears at 1726 cm^−1^, which is attributed to the stretching of the –COO– group, indicating the formation of covalent ester bonds [13].

Considered complementary to FTIR spectroscopy, which is based on the absorption of light with a change in the dipole moment of molecules, Raman spectroscopy is based on the scattering of light with a change in polarizability. When samples are excited by high-intensity laser light, the energy difference between the incident light and the scattered light leads to a Raman shift that is represented as the horizontal axis of the Raman spectrum. Raman spectroscopy is usually employed to characterize the shuttle effect of lithium–sulfur batteries via Raman shift. No Raman shift of the composite electrode occurs if the binder prevents the degradation of electrode and inhibits the shuttle effect [14].

X-ray photoelectron spectroscopy (XPS) is used to measure the elemental composition of a material’s surface and determines the chemical state and binding energy of the elements that compose it. An electron can be ejected when an atom or molecule within the solid surface absorbs an X-ray photon. By counting ejected electrons with different kinetic energies, a photoelectron spectrum with peaks is recorded. These peaks allow the identification and quantification of surface elements. XPS is used to study electrochemical changes in metal-ion battery electrode and binder materials. The electrochemical stability of binders can be characterized by analyzing the XPS spectra before and after electrochemical cycling. A lack of change in the intensity or position of XPS peaks after cycling is an indicator of binder stability [15].

Thermogravimetric analysis (TGA) is used to determine the thermal stability and fraction of components of materials by monitoring the weight change against time or temperature in a controlled furnace. TGA determines the content ratio and thermal stability of binders via the weight loss curve because each binder will typically have a different decomposition temperature [8]. TGA continuously measures the mass of a sample while the temperature is increased over time. When the sample begins to degrade, a mass loss can be recorded, depending on the kinetics of decomposition and the volatility of any residues that are formed.

### 2.2. Analyzing Electrode Microstructure

Scanning electron microscopy (SEM) is a characterization technique used to construct images with topographical and compositional information. Imaging by collecting secondary electrons (secondary electron mode) shows more contrast for sample topography, while collecting backscattered electrons can provide information on the local composition, since higher molecular weight elements scatter a significantly higher fraction of electrons. State-of-the-art scanning electron microscopes can resolve nanometer-scale features and can be used to image contacts between the electrode materials and binder. For example, studies have used SEM to determine the wetting of the binder onto the active material and the effect on homogeneity when adding conductive material [16].

Energy-dispersive X-ray spectroscopy (EDS) is typically used alongside SEM to show the dispersion of various elements within a sample. When the SEM’s electron beam bombards a sample, the electrons, initially in the ground state, are excited to a higher energy shell. This excited state can radiatively return to the ground state by emitting an X-ray with an energy characteristic of the element’s unique electronic structure. This is used to identify and quantify the composition of all elements within the control volume irradiated by the electron beam. For example, binders that have functional groups with distinct elements (e.g., halides) can be discerned from the carbon-containing conductive material via mapping of this element by rastering or scanning the electron beam over the sample. The dispersion of the binder within the coating can be used to predict its success in cementing the electrode together. Thus, SEM-EDS can be used to visualize the electrode microstructure. Post-mortem imaging of cycled electrodes can also reveal if cracks formed due to lithiation-induced volume changes, which would lead to a loss of electrical contact and is a direct failure of the binding material [17].

Photoelectron emission microscopy (PEEM) uses local variations in electron emission that arise via the photoelectric effect to generate contrast within an image. This technique can identify different chemical environments and bonds formed and visualize their distribution. This technique can be coupled with X-ray absorption spectroscopy (XAS), which can provide information on the materials’ oxidation state and chemical environment. This is complimentary to XPS but with higher resolution and typically requiring high-energy synchrotron-generated X-ray sources. Wang et al. [18] used this combination to visualize the elemental composition of cycled electrodes. They were able to deconvolute the F K-edge signal from the poly(vinylidene difluoride) (PVDF) binder and the Li-F bond formed due to electrolyte degradation. This is a powerful tool to use when accurately comparing the microstructure before and after cycling. Since it can distinguish between chemical environments, this technique can be extended to studying binder distribution when the binder is a hydrocarbon and has no distinct element to differentiate it from the conductive or active material, which is a limitation of EDS. To date, this has been done to differentiate between hydrophilic styrene butadiene rubber (SBR) (modified with oxygen functional groups) from the oxygen-containing active material and the carbon-based conductive material [19] and assess binder distribution within the composite coating.

### 2.3. Electrochemical Parameters and Characterization

As previously discussed, polymeric binders are used to improve interparticle contact and adhesion between the electrode coating and the current collector. Ideally, the optimal binder will not degrade in the required potential range, can maintain particle contact, and, therefore, a low interparticle resistance and low electrode polarization. It should also efficiently maintain adhesion between the coating and current collector. Thus, electrochemical techniques used to study binder behavior focus on observing its electrochemical stability, internal resistance within a composite electrode, charge transport, and long-term cycling.

**Figure 3:**
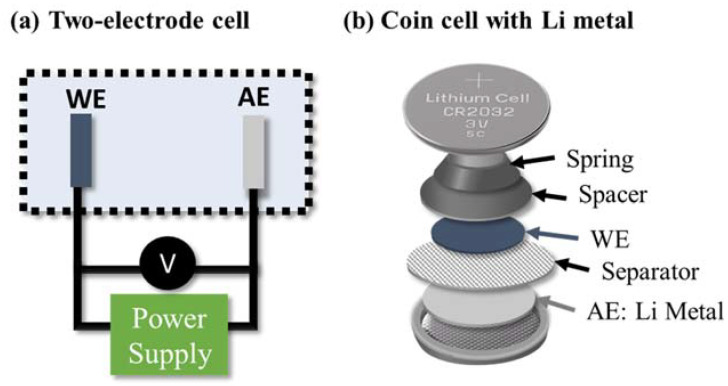
A schematic showing (**a**) a two-electrode setup and (**b**) the composition of a conventionally used coin-type half cell with lithium metal.

The electrochemical characterization of battery materials is often carried out using two-electrode cells consisting of a working electrode (WE, the electrode that is to be analyzed) and a counter or auxiliary electrode (AE), as shown in Figure 3a. In this case, lithium metal is often used as the auxiliary electrode (and it is referred to as a half cell) since it has a small overpotential and stable voltage over a large electrochemical window. Thus, it can be assumed that the response from the lithium metal is negligible, so the overall result is representative of the working electrode. Furthermore, two-electrode systems mimic battery conditions and therefore provide a realistic representation of commercial batteries as they do not contain reference electrodes. Half cells can be constructed as coin cells (shown in Figure 3b), pouch cells, and Swagelok cells [20].

In the cases where specific reactions at the WE must be studied (meaning the WE signal must be separated), then a three-electrode setup should be used. This setup uses an additional electrode referred to as the reference electrode (RE). Conventional reference electrodes are silver/silver chloride (Ag/AgCl) and the saturated calomel electrode (Hg/Hg_2_Cl_2_), which are capable of maintaining a stable voltage during the electrochemical measurement [21]. However, pseudo reference micro electrodes such as silver, platinum, lithium alloy, and gold wires may also be used [22,23]. Another way to separate the WE from AE signals is by using two-electrode symmetrical cells where the WE and AE are identical electrodes at the same state of charge [24].

One technique for examining electrical properties of electrode materials is broadband dielectric spectroscopy (BDS), which enables the investigation of these properties at multiple length scales (including clusters, individual particles, and atoms). This is accomplished by applying a time-dependent electric field to the materials, which produces charge density fluctuations. For frequencies below 10^11^ Hz, these fluctuations result in dielectric relaxations, the sum of which compose the overall dielectric spectra for the investigated solid compounds. Polarizations involving dielectric relaxations can be seen at various frequencies and depend on both the length scale (a response has a lower frequency for larger length scales of the system, such as clusters vs. particles) and charge carrier mobility (with lower frequency for lower mobility, such as ions vs. electrons). As such, scanning over a broad frequency range (typically within the 10 Hz to 10 GHz range) can give valuable information on the electronic or ionic conductivity of an electrode at multiple length scales, as well as at different temperatures and pressures [25]. BDS has been used for applications such as comparing ionic conductivities of polymer binders in Li-S batteries [5] and determining the primary limitations in electronic transport in nanocomposite materials [26].

#### 2.3.1. Cyclic Voltammetry

Using an instrument known as a potentiostat, the voltage across an electrochemical cell can be varied linearly with time to measure the current response. At the end of the scan, the voltage is reversed to give information on both reduction and oxidation half reactions—this technique is referred to as cyclic voltammetry (CV) and is analogous to a battery charge/discharge cycle. Non-zero currents in CV are attributed to non-faradaic responses or capacitive accumulation of electrical charges on or near the electrode surface. Current peaks are indicative of faradaic reactions where the peak position is the oxidation or reduction potential [27].

CV analysis can be effectively used to study the electrochemical response of materials within the operating voltage window. For the polymeric binder, this can be used to determine the stability if no decomposition reactions are observed. This may be accomplished by coating the pristine polymer onto the current collector and assembling a two-electrode cell with lithium metal [28,29,30]. Then, cyclic voltammograms for several cycles can be collected to determine if the binder contributes irreversibly to the faradaic current response, which could indicate degradation over the course of cycling. Moreover, reactions can also indicate contribution toward lithium storage (excess capacity) [31] or irreversible lithium doping of the polymer. The latter, in the case of some binders, has been shown to result in improved conductivity [30,32,33]. Similarly, the electrochemical stability of the binder at varying temperatures can also be determined through CV [34].

The performance of the binder must also be tested within a composite electrode alongside the active and conductive components. Using the Randles–Sevcik equation, voltammograms at varying sweep rates can be used to calculate lithium diffusion within the coating for charge and discharge processes [35]. When compared to an electrode with the conventional binder, this can be used to show the effect of the new binder on intercalation kinetics [36]. Furthermore, peak positions (specifically inter-peak distance between oxidation and reduction peaks) are indicative of system polarization (e.g., kinetic or mass transfer overpotentials) and resistance.

#### 2.3.2. Galvanostatic Techniques

Electrochemical impedance spectroscopy (EIS) is an analytical method used to study the various resistive and capacitive properties of the electrochemical system as a function of frequency (i.e., rate). This technique employs a potentiostat to apply alternating potential at varying frequencies to the system and measures the alternating current, phase shift, and amplitude changes. The varying frequencies allows for the separation and observation of processes that occur in slow or fast timescales [37]. The results acquired can be interpreted using equivalent circuits built by identifying sources of resistance, inductance, and capacitance within the system. The resulting analysis in the form of Nyquist or Bode plots, alongside the circuit, can be used to compare electron transfer resistance (often abbreviated as R_CT_) at high-medium frequencies and lithium ion diffusion at low frequencies [33,36]. This analysis would be complementary to CV.

Often, EIS measurements are carried out on pristine and cycled composite electrodes to determine the change in resistance over cycling. The binding capabilities can be determined as a function of interparticle charge transfer resistance, where an efficient binder can maintain a stable interface and a robust, dense, and electrically conductive network, thereby resulting in a low interparticle charge transfer resistance increase over time. It is important to note that during cycling, the electrolyte may decompose and deposit a solid layer onto the electrode called the solid electrolyte interface (SEI)—this would present itself as another source of resistance (abbreviated as R_SEI_) in the high-frequency region after cycling. A capable binder would allow for the retention of a dense film that could mitigate the thickness of the SEI formed [29,38]. Furthermore, EIS can also be used to model the interfacial resistance at the current collector/electrode interface, which would be observed in the high-frequency region of Nyquist plots [39]. An analysis of the binder’s effectiveness in improving electrode adhesion to the current collector can be made using this information, particularly over the course of cycling. Studying interfacial interactions between the active material particles and binder is particularly important when considering hydrogels [40,41,42]. Polymer hydrogels can offer a 3D porous conducting framework, and EIS and CV measurements can provide insight into the charge transport within these frameworks.

Morasch et al. [43] studied the effect of binder gradients within graphite electrodes via EIS measurements. They prepared composite coatings with varying binder gradients (controlled via the drying stage temperature and confirmed through EDS) and assembled symmetrical cells. Due to capillary forces, the binder migrates and accumulates at the electrode/electrolyte interface. It acts as a pore blocker and increases the electrode tortuosity near the surface, which diminishes ion transport and reduces adherence to the current collector. They used a “blocking electrolyte” that contains ions incapable of intercalating within the coating to measure the response from the surface. Depending on the degree of the gradient, this will translate to a higher measured resistance in Nyquist plots and more pronounced phase angle minima in the Bode plots. This could be a useful tool in measuring binder homogeneity and dispersion as a result of mobility during the drying process and would complement EDS data.

Galvanostatic charging and discharging of the assembled half cells composed of the composite electrodes vs. lithium metal will provide a deep insight into the performance of the binders in a battery environment. In this technique, the half cells are (de)lithiated between their working potential range, and the capacity achieved is measured. The charge/discharge current used is calculated from the theoretical capacity of the active material present. Then, the measured capacity is expressed in ampere-hour and conventionally normalized against the mass of the active material present or the electrode area. This technique will give the measured capacity over time/cycles, charge/discharge voltage profiles, and Coulombic efficiency.

Battery degradation is directly observed through measuring the capacity over various cycles (long-term cycling). Degradation mechanisms are commonly attributed to the loss of cyclable lithium due to SEI formation, the increase of internal battery resistance, structural changes, and mechanical degradation (the pulverization of active material or formation of electrode cracks) [44]. The binder is directly responsible for maintaining electrode integrity. Failure of the binder may result in cracks forming due to internal stress caused by volume changes during lithiation. This would lead to a loss of interparticle contact, electrical isolation of the active material, and result in a significant decrease of capacity, particularly within the initial cycles [31,36,42,45,46,47,48,49].

## 3. Typical Binders for Electrodes

### 3.1. Conventional vs. Aqueous Binders

Most binders used in Li-ion and other batteries are fluorine-containing polymers, particularly poly(vinylidene difluoride) (PVDF). PVDF is the most widely used binder, as it has good chemical and electrochemical stability, as well as reasonable processability. However, it has several disadvantages that have been leading researchers to investigate alternatives. A primary issue is its poor binding affinity to the electrode components. This is because its primary binding mechanisms are mechanical interlocking and van der Waals forces, which are weak and general interactions [1,9,59]. While this has a negative effect on standard Li-ion batteries (with higher affinities from other binders leading to improved performance [60,61]), it is a much larger problem for more recent developments in high-capacity energy storage, such as Si-based anodes. These materials experience a large change in volume during charge/discharge cycles. To be able to withstand the resulting forces and hold the cell together, binders need much stronger interactions with other electrode components and current collectors [62,63]. Additionally, PVDF is electrically insulating, requiring conductive additives for proper electrode function [64]. Another issue of importance is that PVDF requires the use of the toxic organic solvent, *N*-methyl-2-pyrrolidone (NMP), to be processed for electrode fabrication. In addition to the health hazards from this solvent, NMP is also expensive (1–3 $/kg, on top of the relatively high cost of PVDF itself of 8–10 $/kg [65]) and has a high boiling point of about 203 °C, requiring more energy and higher temperatures for its removal during electrode fabrication. Furthermore, while PVDF is used for its chemical stability and does not react with electrolyte, it can swell or dissolve on exposure to electrolyte solvent, leading to capacity loss of the cell [66].

Binders that can be processed in aqueous conditions are able to resolve many of the above issues with conventional binders, with a summary of these binders and their advantages presented in Table 2. Many advantages of these binders arise from the use of water as the solvent. Water is an inexpensive solvent, especially compared to NMP (around 0.015 $/kg for water versus 1–3 $/kg for NMP [65]); these savings add together with the frequently lower cost for commonly used aqueous binders, which often range from 2 to 5 $/kg. Furthermore, using water greatly reduces the environmental impact of electrode fabrication by replacing the use of NMP; as added benefits, this also lowers the emission of CO_2_ equivalents and further reduces fabrication costs, as costly solvent recovery is not required [7,65,67]. Water also has a significantly lower boiling point than NMP, which can speed up evaporation during fabrication.

Other advantages are obtained because of the typical properties of binders that can be processed in aqueous conditions. As mentioned above, commonly used aqueous binders are less expensive than PVDF. Additionally, many aqueous binders repel electrolytes and will not swell upon exposure, helping to maintain cell capacity [62,68,69]. Finally, the functional groups present on actively researched aqueous binders provide stronger interactions with other components; this can improve the stability and longevity of the electrode and potentially reduce the amount of binder required, increasing the proportion of active material to improve energy density.

### 3.2. Current Aqueous Binders

Due to water’s low cost and environmental friendliness, there has been a movement toward the safer and more sustainable fabrication of energy storage devices using aqueous dispersions. One conventional aqueous binder is poly(tetrafluoroethylene) (PTFE), which is applied as an emulsion in water, and it is used either directly or in its sulfonated ionomer form, Nafion [67,70]. While not often utilized for battery electrodes, it is more commonly used to bind high surface area materials in supercapacitors [56,71,72]. The use of spherical PTFE particles leads to a reduced area of interfacial contact. Combined with their ≈100–200 nm size preventing binder penetration into meso- and micropores, PTFE is less vulnerable than PVDF to depletion, which results from adsorption to high surface area materials, blocking the electrochemically active surface area.

One of the primary binders that has seen use in aqueous electrode fabrication is carboxymethyl cellulose (CMC), which is a water-soluble alternative to cellulose that has seen previous use in electrode processing as a thickener to support dispersion and modulate viscosity [65]. CMC was first proposed as a binder for Li-ion batteries by Drofenik et al. in 2003 [60], who found that it performed at least as well as PVDF. In addition, only around 2 wt % of CMC binder was required to give acceptable anode properties, compared with the 5–10 wt % typical for conventional polymeric binders.

In addition to its solubility in water, which facilitates aqueous processing, CMC has other advantages as a binder compared with PVDF. First, it is significantly less expensive, especially when considering the cost of solvent. Second, it has low solubility in commonly used electrolytes, leading to less swelling and little loss of mechanical properties. Third, its biodegradability makes it easier and safer to dispose of. Finally (and most relevant to performance), it offers a greater binding capability for typical electrodes.

The stronger binding of CMC originates primarily from its hydroxyl and carboxyl groups, which allow for greater interactions to materials such as graphite anodes. These groups are able to engage in hydrogen and covalent bonds with surfaces, depending on the specific electrode material. This makes CMC particularly suitable for materials such as Si, where it can form hydrogen bonds with hydroxyl groups on their surface. Importantly, the capabilities of CMC as a binder depend on its structural characteristics, such as the degree of substitution and molecular weight, as well as the environment, such as pH. For example, Mazouzi et al. [73] reported that the binding performance of CMC on Si-based electrodes was greatly improved when the electrodes were processed using an aqueous solution buffered to pH 3. These conditions enabled the formation of ester bonds between the CMC binder and Si particles, which greatly increased the overall interaction strength. As another example, Hochgatterer et al. [74] found that the degree of substitution of CMC, which is how many carboxymethyl groups are substituted per monomeric unit, played an important role in battery performance. CMC with a higher degree of substitution showed superior cycling performance due to the larger number of interactions with the Si particles, binding to more of them and forming a stronger network.

While CMC shows improvements over PVDF as a binder by itself, a popular strategy to further increase its capabilities is incorporating styrene butadiene rubber (SBR) as well. SBR offers more flexibility and a high heat resistance to the resulting binder system, and it also possesses a higher binding force than conventional PVDF [61]. However, Lee et al. [75] found that SBR by itself was unlikely to interact with hydrophobic graphite particles in fabricating anodes, leading to unstable suspensions during preparation. Jeschull et al. [76] investigated CMC-SBR binder at lower, more practical binder contents at both the laboratory and pilot scales, where they demonstrated more consistent improved behavior than alternatives. Together, CMC-SBR binders have improved dispersive capabilities, flexibility, and binding strength, making them a popular choice for aqueous binder [36,77].

With the carboxyl groups in CMC being particularly capable of stronger interactions, other polymers with these groups have also been investigated, notably poly(acrylic acid) (PAA) and sodium alginate. While PAA first found use in electrodes as a dispersant to assist in stabilizing aqueous suspensions (in the form of PAA-NH_4_) [78], Ui et al. [79] demonstrated its use as a binder in graphite electrodes, where it showed superior electrode characteristics to PVDF binder. Additionally, PAA enabled reversible Li^+^ ion intercalation for charge/discharge in pure propylene carbonate electrolyte, which could not be achieved with PVDF. Magasinski et al. [62] extended the usage of PAA binder to Si-based anodes, which typically offer high specific capacity at the cost of significant volume changes over charge/discharge cycles [3]. Investigating PVDF, CMC, and PAA as a binder, they found a significantly improved performance of CMC over traditional PVDF, with PAA demonstrating even greater improvements in stability and performance. As seen in these results, PAA and CMC share several advantages over PVDF, particularly water processability, increased stiffness, and low swelling in exposure to common electrolytes. Compared with CMC itself, PAA possesses higher concentrations of highly polar carboxylic groups, allowing it to engage more strongly in both hydrogen and covalent bonding with materials such as silicon.

For Si anodes in particular, PAA offers further benefits beyond its stronger binding capabilities. Parikh et al. [54] and Browning et al. [80] investigated the role that PAA plays in another important part of the battery: the solid electrolyte interphase (SEI), which forms on the surface of the anode and acts as a passivating layer. The composition and stability of the SEI are important for avoiding irreversible capacity loss of the battery. Parikh et al. proposed that the higher hydrogen bonding strength and polarity of –COOH groups in PAA could accelerate the reduction of ionic liquid electrolyte to LiF and sulfates. They found that the resulting increased bonding strength and polarity led to a faster percolation of sulfates to the surface of Si nanoparticles, where they could be reduced to sulfides with a passivating effect; together, these led to an effective and stable SEI layer that improved cell performance and capacity retention. Similarly, Browning et al. studied SEI formation with PAA binder, and their results indicated that PAA-mediated SEI formation modified the SEI chemical composition and thickness.

Another alternative to CMC with increased binding capability is sodium alginate. Alginate is a natural polysaccharide that is a major component of the cell walls of brown algae. It is actually a copolymer of D-mannuronic acid (M) and L-guluronic acid (G) residues; the ratio and distribution of blocks of M and G residues give different alginates a variety of properties, including mechanical stiffness and the number of ionic crosslinking sites (present in G blocks). Unlike CMC, alginate contains carboxylic groups on each monomeric unit (both M and G), leading to a high concentration of these groups, which bring their favorable properties for binding. Alginate has commonly been used by the food and medical industries as a thickener or as a gel for encapsulation or as a tissue scaffold [81,82,83]. Kovalenko et al. [12] first applied alginate as a binder for Si anodes in Li-ion batteries. They found that alginate as a binder enabled stable performance of the electrodes, which PVDF and CMC did not achieve. The higher polarity of the alginate macromolecules and even distribution of carboxylic groups along their chains led to stronger interactions with the Si nanoparticles and higher adhesion of the electrode to the substrate. Furthermore, alginate was still able to provide ion transport due to its uniform distribution of carboxylic groups, which would support Li-ion hopping between the adjacent sites.

Since the initial report by Kovalenko et al., other works have utilized alginate as a binder for electrodes. One primary advancement of these works is to incorporate a crosslinker for alginate into the slurry for fabricating the electrode. Alginate can be crosslinked noncovalently through multivalent cations, with Ca^2+^ the most commonly used throughout its various applications. Liu et al. [84] added Ca^2+^ ions in the form of CaCl_2_ to alginate binder, combining this with Si–C composite active material and carbon black additive. This improved the mechanical properties of the alginate binder, strengthening the interactions of the chains between each other and improving the tensile strength by 1.77 times. With this improved mechanical strength, the crosslinked gel binder was able to prevent the pulverization or fracture of the electrode over the large volume expansion of the Si–C composite. While Ca^2+^ ions offered improvements, Wu et al. [40] investigated other multivalent cations for use in crosslinking alginate binder for Si anodes, including trivalent Al^3+^. Al^3+^ and Ba^2+^ were found to offer superior mechanical properties to the binder, leading to a longer cycle life and greater capacity retention for their respective electrodes. Alginate has found use in other battery systems as well, such as its use by Ling et al. [69] in sodium ion batteries as a binder for TiO_2_ anodes, where it offered numerous benefits to Coulombic efficiency and retention of charge capacity.

Other works have focused on improving the binding strength of alginate to the active material and the substrate. Gendensuren et al. [85] grafted alginate with polyacrylamide (PAAm) to form a dual-crosslinked network binder. This greatly improved adhesion as characterized by 180° peeling tests on the electrodes, which is likely due to additional binding sites from the resulting branched structure of the grafted polymers. Overall mechanical properties were also improved, which agrees with studies of double network hydrogels such as alginate and PAAm studied by Sun et al. [86]. More recently, Gendensuren et al. [87] sulfonated the alginate in their grafted network, further improving the adhesion and mechanical properties as well as increasing the ionic conductivity of the binder and overall electrode.

Another polymer that could be used as an aqueous binder in electrodes is polyurethane (PU). Polyurethanes are a versatile class of polymer that have several forms, including water-based PU, which is a colloidal system consisting of PU particles dispersed in water. PU can contain a large variety of functional groups, including urethanes, aliphatic hydrocarbons, esters, ethers, and urea, allowing it to be easily incorporated either chemically or physically with other polymers. PU has several advantages for its use as hybrid aqueous binders: (1) Due to its segmented structure with hard and soft domains, PU shows higher elasticity, tensile strength, and adhesion compared to common aqueous binders such as CMC, PAA, or poly(ethylene glycol) (PEG); it can also form hydrogen bonds and physically crosslinked networks with these polymers to enhance their mechanical properties [13,88]. (2) With abundant polar groups such as urethane and carboxyl groups, PU can help to inhibit the shuttle effect in Li-S batteries [53]. (3) PU can encapsulate the active material while allowing the crossover of electrolyte ions, reducing corrosion of the electrode [8].

Despite these advantages, only a few authors have reported on the use of PU as an aqueous co-binder. Zheng et al. [53] used a composite binder consisting of waterborne PU, PAA, and graphene (for improved conductivity). The polyether portion of PU could capture Li ions and accelerate their migration, and the physical crosslinking between PU and PAA greatly increased the elasticity of the binder, which helped to alleviate the large volume change during charge/discharge cycles in Li-S batteries. Loeffler et al. [8] used PU with CMC as a binder for oxide-based cathodes to improve the electrochemical stability and reduce the corrosion of the current collector. In addition, they found that this composite binder in both the cathode and anode electrodes also led to Li-ion batteries with high Coulombic efficiency (≈99.9%) and high capacity retention over many cycles.

### 3.3. Latest Advances in Aqueous Polymer Binders

With the constant development of new battery systems and the demand for higher energy densities and better lifetimes, novel binder polymers are continuously being developed and investigated. For example, Yi et al. [94] have developed an aqueous-processable polymer, chitosan sulfate ethylamide glycinamide (CSEG), by conjugating acryloyl glycinamide to chitosan sulfate to combine the benefits from these two components. The sulfate group of chitosan sulfate improved the aqueous solubility and helped to trap lithium disulfides to prevent their dissolution into electrolyte and protect from the shuttle effect in Li-S batteries. The dual amides from acryloyl glycinamide also adsorb strongly to lithium disulfides and furthermore could engage in numerous hydrogen bonds with each other and the other components, improving the mechanical properties of the binder and its adhesion. Together, these properties resulted in sulfur cathodes with high capacity and stable cycling performance.

While all the binders presented in this review are used in the aqueous fabrication of electrodes, truly green or sustainable production is not guaranteed by this. Scalia et al. [57] have developed a binder using tragacanth gum that offers a more green option. Tragacanth is naturally obtained as dried sap from several species of shrub, and it offers a green alternative to other aqueous binders, which may be synthetic or naturally sourced, but with a toxic or non-sustainable production or extraction process. The tragacanth gum-based binder offered good thermal stability and improved specific capacitance, but its primary advantage comes from its fully sustainable sourcing. However, it was found that using tragacanth could partially hinder electrical transport, which may require an additional conductive additive.

Another naturally sourced binder was developed by Hapuarachchi et al. [52] for Si anodes using tapioca starch, which is a natural and inexpensive polysaccharide from cassava roots. The branched structure of the starch allowed for greater flexibility and stress relaxation during drying. It also led to a greater concentration of hydroxyl groups for hydrogen bonding to the Si active material, which could reform during extreme volume changes of Si. Furthermore, PEG was incorporated into the tapioca starch binder to improve ionic conductivity. However, this did lead to some electrolyte uptake, which could lead to swelling and damage of the binder, requiring tight control of PEG quantities.

While some binder developments have primarily improved sustainability, others have been focused on increasing functionality, often with specific electrode systems in mind. Dong et al. [95] presented poly(methyl vinyl ether-*alt*-lithium maleic acid) (P(MVE-LMA)) as an aqueous binder for use in high energy density LiNi_0.5_Mn_1.5_O_4_ (LNMO) cathodes. P(MVE-LMA) was chosen to solve a key problem with LNMO cathodes, where cyclability suffers from transition metal cation dissolution during operation at high voltages. The lithium carboxyl groups were able to interact with these cations to prevent dissolution, as well as offering strong interactions with LNMO and the current collector for adhesion and cohesion of the electrode. The ether groups assisted with performance by their ability to adsorb on carbon black particles, improving their distribution in the system. Furthermore, the P(MVE-LMA) binder supports the formation of a stable cathode electrolyte interphase, helping to protect the LNMO particles. This work highlights the importance of considering electrode properties and challenges when choosing or developing a binder.

Hwa et al. [7] have developed a new aqueous binder for Li-S batteries based on perylene bisimide, where four carboxylic acid moieties are introduced to the imide positions and subsequently lithiated. This binder consists of molecular subunits of perylene bisimide that self-assemble through π–π stacking. This supramolecular structure is better able to withstand the large expansion and contraction of S active material (similar to that in Si electrodes). The lithiated carboxylic acid groups provided good water solubility and distribution of the binder in aqueous solution and during drying. The resulting weblike nanostructure that formed offered stronger physical binding. Furthermore, these lithiated carboxylic acids were able to trap lithium disulfides to prevent their dissolution into electrolyte, protecting the electrode from the shuttle effect that hinders the usage of Li-S batteries.

Numerous works have utilized mussel-inspired polymers to increase the functionality of aqueous binders due to their strong and reversible adhesion [29,96], which can be incorporated into polymers with additional capabilities, such as conductivity [97]. Mussel-inspired polymeric binders have found particular use with Si nanoparticle anodes; the Si nanoparticles experience extreme volume changes (up to 300% volume expansion) during charging/discharging, requiring binders with high strength [98] or self-healing capability [29].

One important direction for new binders has been the incorporation of self-healing capabilities [99]. This functionality allows electrodes to better withstand the strain of large volume changes during charge/discharge cycles, as well as repairing damage from the movement or impacts in flexible electronics. Achieving self-healing capability for binders often involves both the formation of bonds that are strong, yet reversible, as well as the incorporation of sufficient mobility or flexibility for bonding sites to reform. For example, Jeong and Choi [29] utilized metal–ligand coordination bonding to form strong and reversible crosslinking sites between an Fe^3+^ ion and three catechol groups. Importantly, butyl acrylate monomer units were also incorporated into the polymer to increase its flexibility, improving the efficiency of coordination bond recovery. Jiao et al. [13] used the dynamic exchange of disulfide bonds to incorporate strong, reversible bonds into an aqueous binder. The disulfide group came from bis(4-hydroxyphenyl) disulfide, which was used as a chain extender in the formation of bifunctional polyurethane. This polyurethane offered flexibility and reversible bond formation, which combined with PAA to enhance adhesion and dissipate stress, resulting in a final binder that retained above 88% capacity over 200 cycles. Wang et al. [100] utilized supramolecular chemistry to achieve a self-healing polymer for Li-S batteries. Methacrylated soy protein isolate was used as a macro-crosslink site for polyacrylamide, where numerous amide groups were available for hydrogen bonding. These bonds could break during expansion on the lithiation of sulfur while other covalent bonds maintained the overall structure; then, they could reform when the sulfur reduced in size. This resulted in a binder that could achieve 80% capacity retention over 350 cycles, and only 0.0545% specific capacity loss per cycle after 400 cycles. These works highlight the importance of interaction strength and reversibility in polymer binders not only for adhesion but for improved mechanical properties as well.

## 4. Concluding Remarks

For conventional electrodes, the most commonly used binders are fluorinated polymers, with PVDF being the standard polymer binder. While PVDF has reasonable chemical stability, it requires expensive and hazardous organic solvents and does not bind particularly strongly due to its reliance on weak van der Waals forces for interactions with other materials. Aqueous binders offer several advantages in fabricating electrodes for energy storage devices, including reduced cost and environmental impact. In particular, many have functional groups such as hydroxyl and carboxyl groups that can engage in stronger interactions with other electrode components. Then, these binders must be investigated to determine the chemical, mechanical, and electrical properties. In this review, common characterization techniques are introduced and described, and numerous aqueous polymer binders have been discussed, with a focus on newly developed binders with additional advantages over purely improved binding. One of the most popular choices for use as aqueous binders has been a combination of CMC and SBR, which offer good mechanical properties and binding capabilities at reduced cost. Other polymers have their own advantages, such as the improved adhesion of PAA and alginate due to more carboxylic groups. Other polymers, such as polyurethanes and catechol-bearing polymers, have not been fully explored but show promising use as aqueous binders resulting from improved adhesion and functionality. For the future, two main paths are visible for the improvement of aqueous binders. The first is a move toward more fully sustainable sources that do not require hazardous or expensive treatments at any stage of fabrication. This can be seen in binders using natural starches and gums. The second path is to incorporate additional functionalities to binders, such as electrical or ionic conductivity and the protection of active materials and electrolyte. This path is generally more present in synthesized polymer or copolymer systems. Ideally, a careful balance should be struck to achieve polymer binders that are green, safe, and practical.

## Figures and Tables

**Table 1:** Common techniques for characterizing polymer binders.

Technique	Purpose	References
Fourier Transform Infrared (FTIR) Spectroscopy	- Confirm chemical structure - Confirm bond formation	[13,50,51,52]
Raman Spectroscopy	- Confirm chemical structure (complementary to FTIR) - Examine shuttle effect in Li-S	[14,29,53]
X-ray Photoelectron Spectroscopy (XPS)	- Measure elemental composition - Investigate chemical states - Examine chemical interactions	[15,50,54,55]
Thermogravimetric analysis (TGA)	- Thermal stability of binder - Examine content of binder by decomposition temperature	[8,53,56,57]
Scanning electron microscopy (SEM) and energy-dispersive X-ray spectroscopy (EDS)	- Image the electrode microstructure and interactions between binding and active/conductive materials - Distinguish and image dispersion of active, conductive, and binding materials within the coating based on individual elemental maps	[16,17]
Photo electron emission spectroscopy (PEEM) and X-ray absorption spectroscopy (XAS)	- Identify the chemical environment of the binder alone and within the composite coating - Identify and visualize the distribution of binder within a composite coating when the binder has no distinguishing element (e.g., a halide).	[18,19]
Broadband dielectric spectroscopy (BDS)	- Compare ionic conductivities of various binder materials - Determine primary limitation on electronic conductivity at multiple length scales - Examine role of particles, clusters, and coatings in overall electronic conductivity	[5,25,26,58]
Cyclic voltammetry (CV)	- Confirm the electrochemical stability of pure binder through observation of irreversible reactions - Determine contributions to lithium ion storage - Determine lithium mobility within structure - Study the binder’s thermal stability in an electrochemical environment	[28,29,30,31,34,36]
Electrochemical impedance spectroscopy (EIS)	- Predict the presence of a concentration gradient of the binder material - Determine the binder’s ability to produce and maintain a dense conductive network by studying the interfacial interactions via measuring of: - The charge transfer resistance within the electrode due to poor contact before and after cycling - The resistance due to formation of an insulating SEI layer	[29,33,36,38,43]
Galvanostatic charging and discharging	- Determine binder performance in battery environment - Observe capacity loss due to binder failure leading to cracks and electrical isolation.	[40,45,51,54,55,56,57,58]

**Table 2:** Common polymer binders, their structures, and advantages.

Binder	Molecular Structure	Advantages	References
Poly(tetrafluoroethylene)	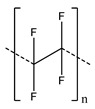	-High chemical and mechanical stability	[56,70,71,72,89,90,91]
Carboxymethylcellulose	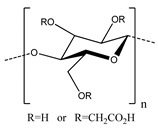	-Inexpensive -Good adhesion -High Young’s modulus	[60,61,73,74,75,76]
Styrene butadiene rubber	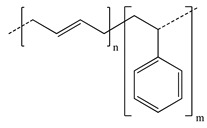	-Good flexibility -High thermal stability	[61,75,76,77]
Poly(acrylic acid)	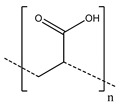	-Controlled structure -Good adhesion	[68,80,92,93]
Sodium alginate	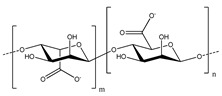	-High adhesion -Good mechanical properties with crosslinking -Properties vary by source	[40,84]
Polyurethanes	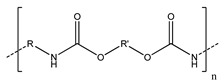	-Can vary functionality -High elasticity and tensile strength -Inhibit shuttle effect and protection from corrosion	[8,53]
Chitosan sulfate ethylamide glycinamide	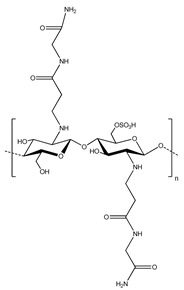	-Protect Li-S batteries from shuttle effect -Good adhesion -Reformable cohesion	[94]
Tragacanth gum (key components shown in structure)	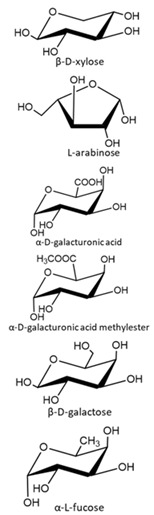	-Fully sustainable source -Good thermal stability	[57]
Tapioca Starch	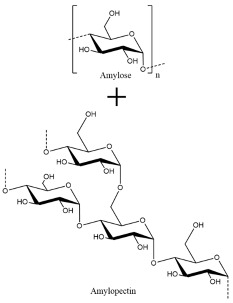	-Inexpensive -Green sourcing -Good flexibility	[52]
Poly(methyl vinyl ether-alt-lithium maleic acid	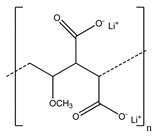	-Prevent transition metal cation dissolution -Good interactions for cohesion and adhesion	[95]
Supramolecular lithiated perylene bisimide	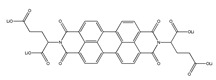	-Can withstand large volume changes -Well-distributed network -Trap lithium sulfides	[7]
